# Case of Congenital Malformation of the Heart

**Published:** 1845-09

**Authors:** John Bell


### RECORD OF MEDICAL SCIENCE.

*Case of Congenital malformation of the Heart.* By JOHN BELL, M. D.—W. A——, the second child of healthy parents, was born 30th of August, 1837. For the first two years of his life he did not manifest any peculiarity of appearance or disorder of function beyond occasional attacks of bronchitis, during which his face assumed a hue seen only in the most violent forms of bronchial congestion. From within eight months after birth until he had entered his fourth year I lost sight of this child.

After again resuming my professional intercourse with the family, my attention was directed to W.'s appearance and sufferings. His face and extremities were then of a dark blue, as were also his lips and tongue; the eyes prominent and shining, and the conjunctiva injected and of a purplish hue; nostrils large and dilated. The sternum and anterior part of the thorax were unduly prominent. The ends of the fingers and toes are broad, swelled and pulpy. Respiration hurried and panting, and the breath emitted during speech with a kind of hissing sound; pulse frequent and rather full, but easily compressed. Impulse and the bellows sound well marked over the sternum. Dulness on percussion was of quite limited extent. The jugulars are much distended. The temperature of the skin, especially at the extremities, is below the natural standard. The appetite was generally good, and the discharges from the bowels and bladder were in normal quantity and regularity.

This boy is easily alarmed, and in the early part of the night often jumps up in affright, as his mother relates; but we may rather suppose that his restlessness proceeds from difficult breathing and an occasional sense of suffocation at this time. His disposition is cheerful, and, unless when suffering from a paroxysm of bronchial congestion, he is mild and easily pleased. He runs about and amuses himself in the same spirits as other children of his age. He lies indifferently on either side. It is not necessary to give the particulars of the repeated attacks of pulmonary oppression and congestion to which this poor little fellow was subjected at irregular intervals, from the time at which I begin this description of his case, in his fourth year, up to that of his death in June, 1845, or two months short of his being eight years old. These were generally induced by the usual causes of catarrh, and sometimes by indigestion. Within the last two years these attacks became more violent, and left him weaker and more irritable and nervous, with less inclination for exercise or attention to his school lessons. The prominence of the sternum and left side of the chest went on increasing; the distention of the jugulars and the pulsation were still more perceptible. The sounds in common were not strong, although those of regurgitation were quite distinct. During a paroxysm, there was, however, a loud bellows and rasping sound, with another less evident, and comparable to a subdued gurgling, or churning, over the sternum. The pulse all the while was frequent but without force. Directly applied over the region of the left ventricle, or to the stethoscope on this part, the ear received the impression of weak sound and movement.

At two different periods, together with great precordial and pulmonary oppression, dyspnœa, &c., there was tympanites, obstinate constipation, and anasarca, against which all the usual diuretics and means of indirect reduction were utterly powerless. Recourse, however, to venesection in quantity varying from two to four ounces, and sometimes cupping, once over the loins, when there was a suspension of the urinary discharge, and, at other times, on the chest, exert an immediate and beneficially controlling influence over the disease, the restoration from which, as far as regards the symptoms enumerated, was as sudden, as it at the time was violent and alarming. The common purgatives, diuretics, and expectorants, would then manifest their customary good effects. Relief was sometimes procured from the wine of colchicum with carbonate of potash, and when the oppression was great, with carbonate of ammonia. Little or no benefit was derived from digitalis. In the two last attacks, the blood was almost entirely destitute of serum, there being not a table-spoonful of the latter to four ounces of the former.

On recovering from the attacks of acute disease, and especially when active treatment had been employed, the skin lost in a great measure its blueness, and approached nearly to its natural colour: but the characteristic expression of the eyes was still preserved. The appetite was generally good, and the craving for various articles, some of them of an indigestible nature, inconveniently great.

The last and longest attack was during the last few days of March and the first three weeks in April of the present year. At this time, the extreme oppression and dyspnœa and other evidences of excessive labour of the heart, coupled with the evidences of malformation of this organ, and the tympanites and anasarca, forbid the hope of recovery, and death was looked for from day to day. Contrary, however, to an apparently evident prognosis, the little patient rallied under the effect of two venesections; the anasarca disappeared, and his feet and legs, at one time enormously swelled, recovered their former size and appearance, and all his functions were restored to their usual condition. He was able to go about the house, and seemed to be, with the exception of weakness, as well as his peculiar state would allow.

On the — of June he was taken out to ride, and he enjoyed greatly the change. On his return, however, he complained of oppression and difficulty of breathing, which rapidly increased, and in a few hours, and before he could be visited by a physician, he was dead.

Unable myself, owing to an accident which confined me to the house, to visit the subject of this case at the time of his death, or to make a *post-mortem* examination, I was fortunate enough to procure the kind offices, on this occasion, of my friend Dr. J. K. Mitchell, by whom, with the assistance of Dr. J. M. Allen, the dissection was made. From Dr. Allen I derive the following notes of the autopsy :—

"Unusual engorgement of the vessels, both superficial and deep seated, with uncoagulated blood. Each pleural cavity contained five to six ounces of serous fluid—the pericardium, a small quantity. Heart very much enlarged, depending entirely on hypertrophy of right side,—the parietes of right ventricle being nearly twice its normal thickness. Nothing peculiar in the appearance of the right auricle. General appearance of the left side of the heart natural, excepting its atrophied condition. An opening about half an inch in diameter at the upper part of ventricular septum, common to the two ventricles and the aorta. Ascending aorta nearly twice its natural size, semilunar valves at its mouth correspondingly large. Ductus arteriosus open, and sufficiently large to admit a goose quill. Pulmonary artery of its natural size at the entrance of the arterial duct, but gradually tapering towards the right ventricle, with which it communicated *directly*, by an opening so small as scarcely to admit the introduction of an ordinary probe. The foramen ovale not entirely closed, but evidently not sufficiently open to allow of any deleterious admixture of venous and arterial blood.

"The only direct communication between the right ventricle and the pulmonary artery, was so small as to escape observation until the artery had been opened and carefully explored. Did this opening at the mouth of the pulmonary artery, during life, transmit blood to or from the right ventricle? The form of the artery and the absence of any valvular arrangement at its mouth, would, it appears to me, favour the former idea."—*Bull. of Med. Science.*

